# Parts, Wholes, and Context in Reading: A Triple Dissociation

**DOI:** 10.1371/journal.pone.0000680

**Published:** 2007-08-01

**Authors:** Denis G. Pelli, Katharine A. Tillman

**Affiliations:** Psychology and Neural Science, New York University, New York, New York, United States of America; University of Minnesota, United States of America

## Abstract

Research in object recognition has tried to distinguish holistic recognition from recognition by parts. One can also guess an object from its context. Words are objects, and how we recognize them is the core question of reading research. Do fast readers rely most on letter-by-letter decoding (i.e., recognition by parts), whole word shape, or sentence context? We manipulated the text to selectively knock out each source of information while sparing the others. Surprisingly, the effects of the knockouts on reading rate reveal a triple dissociation. Each reading process always contributes the same number of words per minute, regardless of whether the other processes are operating.

## Introduction

We take reading to be serial object recognition, where each word is an object. What are the roles of parts, wholes, and context in object recognition? After a hundred years of research into how people identify objects—discrete, nameable, visual stimuli—there seems to be a tentative consensus that the first step is independent feature detection and that the last step is categorization [1]–[5]. What happens in between is less clear. In particular, must the detected features be combined into individual “parts” that must in turn be combined before the object is identified, or is the whole object recognized in one fell swoop? [6].

Many papers on object recognition appeal to the Gestalt notion of the whole being greater than the sum of its parts, but have had only limited success in finding experimental paradigms that bear on that. Experiments using words and faces have found an advantage for identifying whole objects over isolated parts (letters and facial features), which has been taken as evidence for holistic processing, but the effects are not large enough to rule out a solely parts-based process [7], [8]. Other attempts to distinguish holistic from by-parts processing have measured effects of occlusion, scrambling, viewpoint, expertise, inversion, mismatched and misaligned composites, and self-crowding [8]–[15]. Though every study presents data consistent with one process or the other, none of these tests, except scrambling and self-crowding, manages to rule out the alternative [see 16].

Past work has used qualitative tests to choose between holistic and by-parts processes. However, “the distinction between [holistic and by-parts] processing may be a continuum rather than a dichotomy” [17]. Some recognition tasks may benefit from both holistic and by-parts processes. If so, one might ask how much each process contributes. Information from the object's environment and the observer's prior knowledge can be used to recognize objects as well. We lump all task-relevant information other than the object itself into the catch-all “context”. Here we introduce quantitative measures of the contribution of each recognition process: by-parts, holistic, and context.

The question is: if parts, wholes, and context all play roles in object recognition, do the mental processes associated with them interact? Does impairing one process impair the others as well? Or, alternatively, if we remove one process, will the others continue working, unaffected? To explore this question, we turn to reading.

We want to know how people quickly and effortlessly recognize an object when there are a vast number of possibilities. Ordinary reading demonstrates this amazing human skill. In studying object recognition, reading is one of the few cases where one knows the composition: letters are parts, words are wholes, and sentences provide context. Using reading, we can attempt to isolate and measure the contributions of parts, wholes, and context to the recognition of words as objects.

This analysis addresses a central question in reading. What makes fast readers fast, and how should reading be taught to make everyone fast? This question has fuelled a century of reading wars [18], [19]. Each of three processes, which we will call L, W, and S, has been championed at some time, along with a method of reading instruction tailored to emphasize it over the others. L, W, and S each take a different input, but all three processes emit words. Mechanical letter-by-letter decoding, “L”, was once disparaged as fit only for beginning readers. Today it is accepted as the basis for fast adult reading, and schools now teach it through practice in ‘phonics,’ grapheme-to-phoneme conversion [20], [21]. Now consider “S”. Text is somewhat predictable. Readers can predict the next word in a passage 20 to 35% of the time, depending on their reading experience [19], [22]. In the whole-language method, children are encouraged to use the story and sentence context (S) to guess the next word. Lastly, holistic recognition of words by their shape, “W”, once seemed a promising visual account for fast recognition of words, supported by evidence from the Word Superiority Effect (but see ref. 7), and motivated the whole-word method, which had children memorize and read the same few “sight words” over and over. Work on ‘crowding’ has shown that words are not usually recognized as wholes, even by adults, but rather that the visual system must isolate and recognize the individual letters to get the word [8]. However, when the word is crowded, it is impossible to isolate the letters. We call what can still be gleaned ‘word shape’.

Bouma showed that words can only be recognized when the letters are spaced far enough apart [23]. This critical spacing depends on where the word is in the visual field and little else [24]. When the letters are separated by less than the critical spacing, the reader cannot identify them, and the word is illegible. Critical spacing increases in proportion to distance from fixation. For text of any given letter spacing, there is a central field that is uncrowded, and a peripheral field that is crowded [25].

Here we measure the contributions of the L, W, and S processes to reading rate by manipulating text in ways that selectively knock out each source of information while sparing the others. Scrambling word order knocks out the S information, which the reader uses to guess the word from its context:
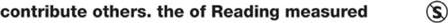



**Figure 1:**
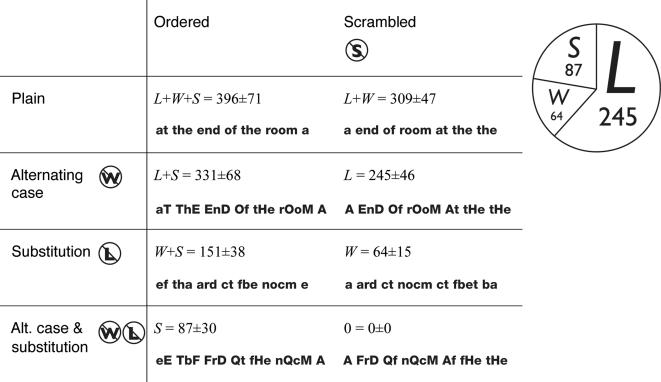
The LWS model of reading rate. The model (Eq. 1) has three parameters (*L, W, S*), the reading rates of the letter-by-letter, whole-word, and sentence-context processes. For each condition, the model predicts a reading rate (word/min) that is the sum of the rates of the spared processes. For each of 11 observers, we measured reading rates for all eight possible combinations of knockouts. The model was fit, separately, to the data for each observer. This table shows the model fit. The observer data are shown in Table 1. The fit's mean±SD, across observers, is shown for each condition. (The SE is about one third the SD.) The model fits every observer well (Table 2). The overall RMS error, across conditions and observers, is 22 word/min. The excellent fit of the additive model (Eq. 1) proves triple dissociation with a combination rule of summation [37].

Alternating case knocks out the W information, which the reader uses to recognize words by their gross shape: 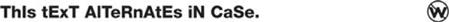



**Figure 2:**
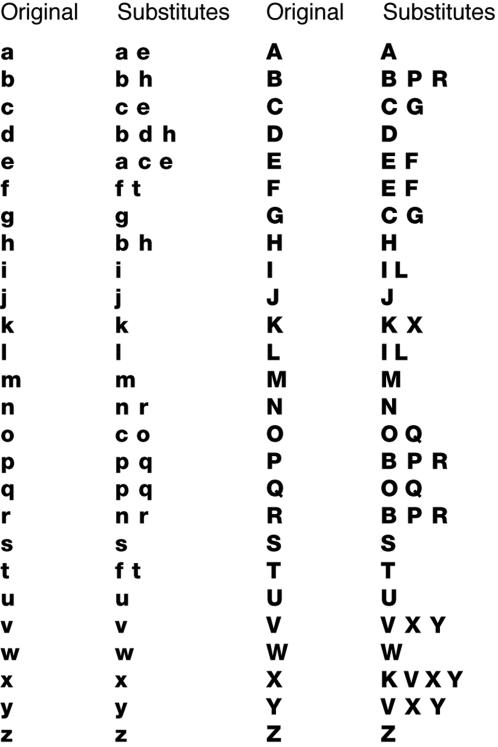
Letter substitutes. Each letter can be randomly replaced by any of its substitutes, including the letter itself. The font is Helvetica Neue LT 85 Heavy from Linotype.

Substituting similar letters (indistinguishable when viewed peripherally) knocks out the L information, which the reader uses to identify the word by identifying its letters:
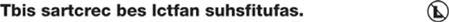



The alternating-case and word-shuffling manipulations are borrowed from the reading literature as-is [26], [27], but we used our knowledge of object recognition to refine the substitution paradigm [28]. As you see, the L knockout is devastating, but we know it spares word shape, as defined here, because the substitutions are undetectable when crowded. Recognizing an object by parts (a word by letters) requires isolation of each part from the rest of the object [24], [29]. When the isolation field is bigger than the word, which happens when the word is far from the center of gaze, the word can only be seen holistically. We use this–what can be seen holistically–as our definition of ‘word shape’. Thus, any two letter strings that are indistinguishable under these conditions have the same word shape.

Using peripheral viewing, we discovered, by trial and error, which letter substitutions could be made without affecting word shape. In the demonstration below, you can verify that our substitutions preserve the word shape of “Reading”, by fixing your eye on the plus and comparing the two words peripherally.
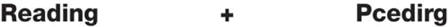



They are indistinguishable even though only 3 of the 7 letters (d, i, and g) are the same. The letter substitutes that passed our test (indistinguishable when viewed in the periphery with flanker letters on both sides) are listed in Figure 2. This list was used for letter substitution.

Alternating case knocks out the holistic word process (W), which can identify some highly familiar words even when the letter (L) information is degraded by crowding or letter substitution. 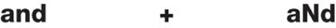



When fixating the plus, the word “and” on the left is obvious, even though the letters are crowded. You are using word shape to read “and”. On the right, you can see that there is a word, and you might even be able to get the exposed letters on the ends [23], but you can't read the word. Alternating case has destroyed word shape. Few words can be recognized by word shape alone, which is consistent with reports that alternating case has at most a small effect on reading speed and accuracy (26, 30–34).

We can be confident that each of these three manipulations affects only one of the three sources of word information in the text. But what about the corresponding recognition processes? The three kinds of information are distinct, but the processes may not be. Can we selectively knock out one process, or does impairing one process impair the others as well? By applying these manipulations one at a time, we measure how much each word-recognition process–L, W, and S–contributes to normal reading rate. By applying them in combination, we test the selectivity of the knockouts and discover the degree to which the reading processes depend on each other.

## Results

We applied every combination of the three knockouts 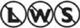 to text from a bestselling Mary Higgins Clark murder mystery [35] and measured reading rate (Figure 1, Tables 1–2). These reading rates were collected using Rapid Serial Visual Presentation (RSVP) [36] in conjunction with a staircase procedure to find the threshold word presentation rate yielding 80% correct accuracy (see Materials and Methods). We also measured rates of both oral and silent reading of printed pages (Table 3).

**Table 1:** Reading rate for each condition for each observer.

Condition		Observer											Mean±SD	
Intact	Knockout	MZ	EK	KT	MM	JC	JB	JS	SP	BR	KB	EG		
L W S	*None*	310	434	486	432	393	424	509	399	339	272	418	401±71	word/min
L S	W	264	364	472	331	276	358	456	372	292	209	324	339±79	word/min
W S	L	135	167	197	160	102	137	193	136	160	96	58	135±38	word/min
S	L W	58	133	154	78	42	110	83	135	95	68	44	91±38	word/min
W L	S	231	292	370	326	278	300	323	311	243	216	314	291±46	word/min
L	W S	202	265	322	243	222	263	269	289	224	190	264	250±39	word/min
W	L S	96	155	104	84	83	119	100	116	99	50	20	93±36	word/min
*None*	L W S	21	110	91	15	34	40	47	78	41	35	40	50±30	word/min

The QUEST staircase procedure homed in on the threshold reading rate to achieve 80% accuracy in each condition [55]. All rates reported here are averages over two or three runs of the same condition. We report SD. SE, with n = 11, is about one third the SD.

**Table 2:** Parameters of the model's fit to each observer's reading rates.

Rate	Observer											Mean±SD	
	MZ	EK	KT	MM	JC	JB	JS	SP	BR	KB	EG		
*L+W+S*	318	437	514	416	355	419	477	422	353	270	371	396±71	word/min
*L*	185	240	310	249	230	253	302	263	196	173	288	245±46	word/min
*W*	67	86	64	84	73	71	59	59	67	46	33	64±15	word/min
*S*	65	111	140	83	52	93	117	100	90	51	50	87±30	word/min
RMS *ε*	16	34	21	8	20	24	36	30	19	11	23	22±9	word/min
*L*/(*L*+*W*+*S*)	0.58	0.55	0.60	0.60	0.65	0.60	0.63	0.62	0.56	0.64	0.78	0.62±0.06	
*W*/(*L*+*W*+*S*)	0.21	0.20	0.12	0.20	0.20	0.17	0.12	0.14	0.19	0.17	0.09	0.16±0.04	
*S*/(*L*+*W*+*S*)	0.21	0.25	0.27	0.20	0.15	0.23	0.25	0.24	0.25	0.19	0.13	0.22±0.04	
RMS *ε*/(*L*+*W*+*S*)	0.05	0.08	0.04	0.02	0.06	0.06	0.08	0.07	0.05	0.04	0.06	0.06±0.02	

**Table 3:** Three ways to measure reading rate.

Rate	Silent page	Oral page	Oral RSVP	Mean±SD	
*L+W+S*	289	251	437	326±98	word/min
*L*	159	140	240	180±53	word/min
*W*	55	29	86	57±29	word/min
*S*	74	82	111	89±19	word/min
RMS *ε*	15	18	34	22±10	word/min
*L*/(*L*+*W*+*S*)	0.55	0.56	0.55	0.55±0.01	
*W*/(*L*+*W*+*S*)	0.19	0.11	0.20	0.17±0.05	
*S*/(*L*+*W*+*S*)	0.26	0.33	0.25	0.28±0.04	
RMS *ε*/(*L*+*W*+*S*)	0.05	0.07	0.08	0.07±0.01	

Parameters of the model's fit to observer EK's reading rates for the three kinds of reading.

For every reader tested, for both RSVP and page-reading, a simple additive model,
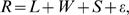
(1)provides an excellent fit to the 8 reading rates, where *R* is reading rate (word/min), *L, W*, and *S* are the observer-dependent reading rate contributions of the three sources of information, each assumed to be zero when knocked out, and *ε* is the error in the fit. Across conditions and readers, the RMS error is a mere 22 word/min (out of a total rate of 396 word/min with no knock-outs). The additive model represents triple dissociation with a combination rule of summation [37]. Each knockout zeroes one component without affecting the other two. The excellent fit with large effects and negligible error proves the triple dissociation.

Confirming the psychologists and educators who emphasize phonics, mechanistic letter decoding, L, accounts for the lion's share (62%) of the adult reading rate. This is recognition by parts. Holistic word recognition, W, accounts for only a small fraction (16%) of reading rate. (This is consistent with Smith's report of 21% reduction in reading speed when case is alternated in this way [38].) The contextual sentence process, S, accounts for 22% of reading rate, on average, but is variable across readers (mean±SD = 87±30 word/min), which may reflect individual differences in print exposure (see ref. 19).

A 3-way analysis of variance of each observer's reading rates for the eight conditions (no repeated measure) shows that the main effect of L is significant (p<0.05) for nearly all the observers (10 of 11), and that the main effects of S and W are significant for nearly half of the observers (5 and 4 of 11). Interactions reached significance in only three cases (L*S for 2 of the 11 observers; L*W for 1 observer). Doing one 3-way analysis of variance of all the data, treating the 11 observers' results as repeated measures, finds highly significant (p<0.001) main effects of L, W, and S, a small interaction of L and S (p<0.01), and no other 2- or 3-way interaction (p>0.5). The main effects account for most of the variance: L 75%, S 7.1%, W 3.3%. The L*S interaction accounts for only 1% of the variance. L and S are slightly super-additive, a synergy. The benefit from L and S together is slightly more than the sum of the benefits from just L or S alone. However, this effect is very small–too weak to have any practical consequence–and is no longer statistically significant when we apply Bonferroni correction for 7 hypotheses. Thus, the ANOVA endorses the additive account.

Understanding individual differences in reading rate would be invaluable. The breakdown in Table 2 compares the contributions of each process across observers. There is surprisingly little difference in the contributions of each of the 3 processes across our group of 11 normal readers. However, note that observers JS and KT, our fastest readers, also have the highest percent contribution of the S (context) process. This supports the idea that the context process reflects differences in print exposure [19]. Even so, these readers are fast mostly because their L processes are fast.

## Discussion

Our main result is the discovery of a triple dissociation among L, W, and S. A within-task triple dissociation with a composite measure is evidence that “the task is accomplished by a complex process that contains [three] functionally distinct and separately modifiable parts” [37].

That letters, words, and sentences are all involved in reading is nothing new, but finding that their contributions to reading rate are additive is startling. Even so, our results are consistent with the Gough et al. [22] study that isolated the contributions of word ‘form’ and sentence ‘context’. They measured the proportion of words correctly named, isolating the contribution of form by measuring the effect of word duration and isolating the effect of context by measuring the effect of the number of preceding words in the sentence. They found that “the probability of word recognition given both form and context conforms very closely to the values one would obtain if the contributions of form and context were independent” [22]. Here, we separate ‘form’ into its two components, L and W, and show that the contributions of these processes to reading rate are independent of each other and of the contribution of the sentence context (S).

What do our results say about the mechanisms underlying reading? One might be tempted to think that the additive, independent contributions to reading rate mean that there are three completely autonomous reading processes. But that doesn't follow. It's not that simple. If the three processes were operating independently, most of the words produced by the two weaker processes (S and W) would be redundant with those produced by the stronger process (L). This would mean that the contributions of S and W would be greater when L is absent than when L is present, which is not what we find.

Additivity of rates implies exclusivity. The contribution of each process to reading rate is the same whether the other processes are working or not. Thus, the contributions are not redundant. The three processes are not working on the same words. This requires coordination among the processes. For the contributions of S and W to be equally valuable with and without L, L has to skip those words or devote only a small fraction of the time it normally would to those words before moving on to the next one.

Imagine that L, W, and S are three technicians in a computer store. As customers arrive, the technicians avoid handling the same ones. Instead, L is a generalist, who handles most of the customers, while S and W are specialists, who only handle certain kinds of computer problems. S's and W's total performance cannot match L's, because they only handle certain customers, but, for those customers, they are faster than L would be. When such a customer walks in, S or W immediately lets L know he will handle it, so L can take the next person.

The customers (words) that S and W can handle are infrequent. L could work on all words, but usually does not need to, because S and W handle some of them. To help, S or W must let L know immediately that he will get this word. In the case of S, supposing early notification is supported by MEG and ERP research showing that predictable words are processed much earlier than unpredictable ones [39], [40]. We suspect that W, too, warns L early. It takes time to assemble the parts of a complex word [41]. From this, we might expect the one-step assembly (features to word) of the holistic (W) process to be faster than the two-step assembly (features to letters to word) of the by-parts (L) process.

Word shape has been a slippery concept [42], [43]. Here, defining word shape by a practical test for holistic equivalence allowed us to isolate and measure the contribution of the process, W, that recognizes words as wholes.

Past studies have measured effects of substituting letters [28], [44], changing case [26], [30]–[34], [38], and shuffling words [27], but, applied separately, these manipulations only assess how much each degradation of the text impairs reading. This says something about effectiveness, but nothing about the specificity of the knockout. Only by combining the manipulations could we discover the additivity. The triple dissociation proves that the text manipulations are selective. We hope the newly-revealed selectivity of these time-worn tools will prove useful in further explorations of reading.

Our findings challenge the most successful models of reading. Ever since the discovery that reading consists of a series of fixations rather than a continuous sweep of the eyes across the text [45], investigators have looked to the eye movements for clues into how reading works. E-Z Reader [46] and SWIFT [47] model the eye movements of reading. These models include a word-recognition stage whose latency is affected by language properties such as word familiarity and word length, so they do make some use of context and word shape, but they cannot read at all when the letter information is knocked out. Similarly, Mr. Chips [48] and the Dual Route Cascaded model [49] simulate a wide variety of visual and language effects on reading rate and latency, but both models implement only the L process, plan to later add S, and omit W entirely. Our results cry out for implementation of W and S, which, together, account for 38% of reading rate. We suspect that it will be difficult for the models to achieve the additivity of rates that is so robust in the human data presented here.

Our approach treats reading as serial word recognition. It is surprising that such a simple model succeeds so well in describing how readers benefit from the three sources of information available in reading. Our results affirm the practical emphasis on L in schools, but challenge current computational models, revealing that W and S do contribute and make reading possible when L is knocked out. It has long been known that we recognize objects by parts, wholes, and context. The surprise is that each process contributes the same number of words per minute regardless of whether the others are operating. This is a triple dissociation among parts, wholes, and context.

## Materials and Methods

### Reading rate

We measured reading rate in three ways. The results in Figure 1 and Tables 1–2 used the RSVP method described below. We also collected similar results on one observer (EK) reading printed pages, aloud and silently (Table 3). In the page-reading experiments, the observer was instructed to read each page as quickly as possible while still getting most of the words right. Aloud, she read 80%–100% of the words correctly in every condition. Accuracy could not be measured when she read silently. The LWS model fits well in every case, and the parameters' proportions were very similar for the three methods (Table 3).

Past studies have found that reading rate and comprehension when reading words presented serially (RSVP) are not very different from when reading static words on a page. Yu, Cheung, Legge, and Chung compare reading rate as a function of text size for text presented dynamically, one word at a time (RSVP), or statically, all together (static flashcard with four lines of text) [50]. RSVP reading is faster (1.4×) but the log reading rate curves are parallel (one is shifted upward), showing the same dependence on spacing. Masson assessed readers' comprehension of texts presented using RSVP versus statically [51]. He found that when 500 ms pauses were inserted between sentences, accuracy in answering specific questions about the text read was the same for 500 word/min RSVP as when the whole passage was displayed for the same total length of time [51, Table 2, page 270].

### Observers

Eleven observers (ages 17–25) participated. All had normal or corrected-to-normal vision and were fluent in English. All observers gave informed consent and were paid for their participation. Observer KT is an author.

### Stimulus generation

Our stimuli were generated using MATLAB with the Psychophysics Toolbox [52; 53; http://www.psychtoolbox.org] and were displayed on a Philips FIMI GD402 very high brightness 21” grayscale monitor, sold in the USA by AFP Imaging as the “BrightView”, whose frame rate was 75 Hz. Words were presented as black text on a white background (156 cd/m^2^).

### Text

The text came from the mystery novel *Loves Music, Loves to Dance* by Mary Higgins Clark [35], a bestseller written for a broad, popular audience. It is an easy text, with a 7.5 Fog Index and a 5.5 Fleish-Kincaid Index. The text was not edited in any way before the application of the LWS manipulations. All proper nouns, capitalization (except in alternating-case trials), and punctuation were retained. The text was displayed in the Linotype font Helvetica Neue LT 85 Heavy, at an x-height of 0.39 deg and center-to-center letter spacing of 0.53 deg. This imposes uniform center-to-center spacing, overriding the font's ordinary spacing. No observer read the same passage twice.

### RSVP

Using Rapid Serial Visual Presentation (RSVP), we presented each word, one after the other, in the same place on the screen [36]. The reported reading rate (Table 1) is the rate at which words were presented, six per trial, while the observer achieved an accuracy of 80% correct. On each trial, the observer read the six words aloud, taking as long as she liked. Legge notes that “for procedures in which maximum reading speed is computed from the display time of short texts, and oral reading speed is used only to check for accuracy, oral and silent reading speeds are approximately the same” [54].

Our average reading rate of 396 word/min is faster than the typical reading rate of 250 word/min for adults reading a printed page. Our rates are faster partly because RSVP eliminates the need for eye movements [36] and partly because of a speed-accuracy trade-off. We use an adaptive procedure that iteratively adjusts the presentation rate to achieve a desired error rate. The error rate of normal silent reading is very low and hard to measure. We used the criterion of 80% correct, i.e. 20% mistaken, which allows faster reading.

The controlled error rate (achieved through the adaptive control of presentation rate) is an important feature of our experiment. We want to know how quickly observers can read when pressed. We are not interested in how their preferred rate might be affected by unfamiliar formatting of the text. Instead of leaving it to the observer's whim, the RSVP presentation controls the presentation rate, and the adaptive procedure (QUEST) finds the rate that yields 80% correct identification of the words [55]. The fixed error rate (80% correct) contributes to the specificity of our knockouts. The contribution of the sentence context (S) would be reduced by any condition that reduced the fraction of words identified. Our procedure adjusts presentation rate to maintain a fixed accuracy.

All our reported rates and model fits in Figure 1 and Tables 1–2 were collected with an 80% accuracy criterion. Pilot results using higher and lower criteria are consistently well-fit by the LWS model. The LWS rates decrease as the accuracy increases.

### Spatial and temporal flankers

In order to simulate page reading, where each word is preceded and followed by another word, we added a random letter flanker one letter-space away from the beginning and end of each target word: “x word h”. Observers were asked to ignore the flankers.

In order to minimize end-effects in the 6-word sequence of a trial, we added a random letter string before the first word in a trial and another after the last word. These temporal flankers were displayed for the same length of time as the target words in that trial, and observers were asked to ignore them. Without temporal flankers, the first and last words in a trial showed a strong advantage over the middle four. With the temporal flankers, there is no longer any advantage for the last word in a trial. The primacy effect, enhanced performance on the first word, was reduced, but not eliminated, by the addition of temporal flankers.

### Fixation

Two black squares (0.2 deg) were centered 0.9 deg above and below the center of the word. The observer, seated 200 cm from the screen, was instructed to fixate between the two squares and read the words aloud. Spatial and temporal flankers (as described above) were present on all trials.

### Trial, run, and threshold

Each trial contained six words, presented one at a time at the same location (between the black squares). Each run consisted of 15 trials. Except for the scrambled condition, explained below, the text for each trial and run began at the point in the novel where the previous trial and run ended. Each observer completed approximately ten practice runs with plain text before data collection began. The 8 reading conditions of Figure 1 were tested in random order. Before each run, the observer was told which condition she would be reading. The observer was given unlimited speaking time, and correctly read words were counted regardless of word order. Errors in word order were rare, occurring on less than 10% of the trials, with and without scrambled word order. After each trial, an answer screen showed the correct six words, and the experimenter recorded the number of words missed. Observers were encouraged to look at the correct words on the answer screen. The QUEST adaptive staircase increased or decreased the presentation rate of the words for the next trial, homing in on threshold rate for 80% accuracy [55]. Each reading rate recorded in Table 1 is the average of two or three runs.

### Word shape and letter substitution

The approach we are taking to shape is analogous to the scientific approach to color. When multiple mechanisms are potentially involved in a task, it is useful to design stimuli so that one of the mechanisms can't tell them apart. Then, any effects of exchanging the stimuli can be attributed to the other mechanisms, excluding the one that is blind to the difference. This technique is called “silent substitution” [56]. It is common in color vision experiments to equate the luminance of two stimuli and thereby rule out any role of the achromatic channel in accounting for differences in response to these stimuli.

In the perceptual discrimination test used to generate Figure 2, the observer compared the peripherally-presented original letter and candidate substitute, each flanked on both sides by the same pair of flanking letters and judged whether they were discriminable or not. Letter size was 2 deg x-height, center-to-center spacing was 2.5 deg, and the triplets were presented at horizontal eccentricities of ±10 deg. Note that this paper is not about peripheral vision. Nor did we seek out the periphery as an example of bad vision. This paper is about reading. We use peripheral vision as part of an experimental technique that isolates a shape process (W) that is common to both central and peripheral vision. We are using the periphery in a particular way, to knock out the letter-based process, in order to isolate the word-shape channel. We empirically assembled a table of letter substitutions that are invisible to the observer's perception of word-shape when the letter-based identification mechanism is absent.

When Underwood and McConkie [31] used letter substitution to destroy word shape in their moving window paradigm, they defined “word shape” as the gross outline, and selected substitute letters from the original letter's category: having an ascender (e.g. bh), descender (e.g. pq), or neither (e.g. ac). However, though hallowed by tradition, there is no theoretical or empirical basis for that definition of word shape. We instead define word shape operationally (what can be distinguished when crowded) and choose the letter substitutes so as to be visually indistinguishable from the original when crowded. However, this is less different than it sounds, as our letter substitution table turns out to be similar to theirs.

In the L knockout condition, we explained to observers that letters would only be substituted by other letters that look like them (for example, letters with ascenders would never be substituted by letters without ascenders). Observers were told to say the original word they thought the target had been before substitution, not to try to pronounce nonsense words. Observers were also informed that not all letters have substitutes, and letters with substitutes were not always substituted (i.e., a letter with substitutes could be randomly substituted by itself). Because of these rules, some words in substitution trials were unmodified. Even so, the substitution manipulation was very effective. It slowed reading to a crawl.

The L knockout by substitution is quite effective, but not total. We attribute the residual reading rate (50 word/min) in the triple-knockout condition (see Table 1) to letter decoding, i.e. we think that *L* as reported here slightly underestimates the true value of the process. We tried making the knockout more severe (by not allowing a same-letter substitution when alternatives were available) but, as one would expect, this makes some conditions untestable because the observer cannot reach 80% correct at any presentation rate.

As you might guess from the demonstrations in the Introduction, most words are not easily identified by word shape. Random words cannot be read reliably based on word shape alone [8]. The W process identifies only some of the words. These are often short, high-frequency words such as “and” (see demo in Introduction).

### Scrambling sentences

To knock out the S process, word order was scrambled across the 90 words in a run.

### Preserving sentence context

As mentioned above, each (ordered) trial began where the previous trial left off. This means that only some of the trials began at the beginning of a sentence. Many RSVP reading studies, some of which report extremely high reading rates, begin each trial at the beginning of a sentence [e.g., 27]. However, we do not think our method lessened the contribution of the S process in the ordered trials. First of all, sentence context is preserved by our use of an 80% criterion, which insures that readers recognize most of the words. Also, we always presented the correct six words on the response screen at the end of each trial. Therefore, at the beginning of any trial after the first, the observer had the context of the novel up to that point. Granted, we did randomize the order of scrambled and ordered runs. But still, within any given run with S intact, the observer is given much opportunity to make use of the storyline. This is, we think, more like the normal reading experience than using random sentences or artificially restricting our text to only include 6-word sentences.

### ANOVA

Our analysis of variance (Table 4) used the ANOVAN command in MATLAB.

**Table 4:** Results of 3-way Repeated Measures ANOVA.

Source	Sum of squares	Deg. of freedom	Mean square	*F*	*p*
L	1143040.1	1	1143040.1	457.82	0
W	50432.3	1	50432.3	20.2	0
S	108290.6	1	108290.6	43.37	0
L*W	401.6	1	401.6	0.16	0.6894
L*S	18280.3	1	18280.3	7.32	0.0083
W*S	778.1	1	778.1	0.31	0.5782
L*W*S	609.9	1	609.9	0.24	0.6225
Error	199736	80	2496.7		
Total	1521569	87			

We find highly significant main effects of L, W, and S and a slight interaction between L and S. Applying a Bonferroni correction for seven hypotheses reduces the criterion *p* value from 0.05 to 0.0071, rendering the L*S interaction insignificant.
